# Modelling of a targeted nanotherapeutic ‘stroma’ to deliver the cytokine LIF, or XAV939, a potent inhibitor of Wnt–β-catenin signalling, for use in human fetal dopaminergic grafts in Parkinson’s disease

**DOI:** 10.1242/dmm.015859

**Published:** 2014-08-01

**Authors:** Jing-Wei Zhao, Sean C. Dyson, Christina Kriegel, Pam Tyers, Xiaoling He, Tarek M. Fahmy, Su M. Metcalfe, Roger A. Barker

**Affiliations:** 1 John van Geest Centre for Brain Repair, Addenbrookes Hospital, University of Cambridge, Cambridge, CB2 0PY, UK.; 2 Department of Biomedical Engineering, Yale University, Malone Engineering Center, 55 Prospect Street, New Haven, CT 06511, USA.

**Keywords:** Parkinson’s disease, Nanotherapy, LIF, XAV939

## Abstract

The endogenous reparative capacity of the adult human brain is low, and chronic neurodegenerative disorders of the central nervous system represent one of the greatest areas of unmet clinical need in the developing world. Novel therapeutic strategies to treat them include: (i) growth factor delivery to boost endogenous repair and (ii) replacement cell therapy, including replacing dopaminergic neurons to treat Parkinson’s disease (PD). However, these approaches are restricted not only by rapid degradation of growth factors, but also by the limited availability of cells for transplant and the poor survival of implanted cells that lack the necessary stromal support. We therefore hypothesised that provision of a transient artificial stroma for paracrine delivery of pro-survival factors could overcome both of these issues. Using leukaemia inhibitory factor (LIF) – a proneural, reparative cytokine – formulated as target-specific poly(lactic-co-glycolic acid) (PLGA) nano-particles (LIF-nano-stroma), we discovered that attachment of LIF-nano-stroma to freshly isolated fetal dopaminergic cells improved their survival fourfold: furthermore, *in vivo*, the number of surviving human fetal dopaminergic cells tended to be higher at 3 months after grafting into the striatum of nude rats, compared with controls treated with empty nanoparticles. In addition, we also analysed the effect of a novel nano-stroma incorporating XAV939 (XAV), a potent inhibitor of the developmentally important Wnt–β-catenin signalling pathway, to investigate whether it could also promote the survival and differentiation of human fetal dopaminergic precursors; we found that the numbers of both tyrosine-hydroxylase-positive neurons (a marker of dopaminergic neurons) and total neurons were increased. This is the first demonstration that LIF-nano-stroma and XAV-nano-stroma each have pro-survival effects on human dopaminergic neurons, with potential value for target-specific modulation of neurogenic fate in cell-based therapies for PD.

## INTRODUCTION

The prevalence of neurodegenerative disease is increasing as the population ages. After Alzheimer’s disease, Parkinson’s disease (PD) is the second commonest neurodegenerative disorder of the central nervous system (CNS). In PD, the disease process critically involves the nigrostriatal dopaminergic (DA) neurons, resulting in their loss and subsequent decreased dopamine levels in the dorsal striatum. The concept of replacing lost DA neurons with healthy cells of the same lineage underpins one major clinical approach that uses allografts of human fetal ventral mesencephalic (hfVM) tissue. This strategy has shown significant long-term benefits in some patients with PD (reviewed by Barker et al., 2013). However, the approach has a number of ethical and practical problems inherent to it and, thus, for the future, the derivation of DA precursors from embryonic stem cells or induced pluripotent cells will be an essential key research goal for clinical translation. But, as is the case for all cellular therapeutic approaches, two challenges arise: first, the need to deliver appropriate numbers of cells to the right site(s) and, second, to promote their survival long-term. In this latter respect, therapeutic support of the transplanted cells is required, raising the question, can the delivery of neurogenic growth factors or small molecules improve the efficacy and efficiency of cell therapy? The simplicity of such an approach has been hampered by the problem of delivering sufficient concentrations of factors in a sustained paracrine-type manner locally at the site of implantation. As a potential solution, we have been exploring the development of a biodegradable, surrogate stroma preloaded with a defined cargo of growth factor(s) or small molecules, the stroma itself being created by cargo-loaded solid-phase nano-particles of some 100 nM diameter, attached to the cell surface by cell-specific targeting antibody. Our prototype uses poly(lactic-co-glycolic) acid (PLGA), which is already approved by the FDA for therapeutic devices (Danhier et al., 2012). *In vivo*, PLGA slowly degrades by hydrolysis to lactic acid and glycolic acid, monomeric derivatives that feed into metabolic pathways for eventual release as carbon dioxide and water. PLGA allows for the creation of ‘designer’ particles for delivery; these particles can be modified for specific targeting as well as the type of cargo (which can be multiple) and the release kinetics of that cargo. Other approaches that have used synthetic biomaterials to mimic the extracellular microenvironment include bioengineered fibrillar matrices (reviewed by Lutolf and Hubbell, 2005), or adhesive micro-particles to deliver nerve growth factor (Mahoney and Saltzman, 2001). In the current study, we use nano-particles functionalised to bind directly to individual cells, testing cargos able to influence development in addition to cargos that support neurogenesis.

**RESOURCE IMPACT**

**Background**

Neurodegenerative diseases of the brain represent a large and increasing area of unmet clinical need. After Alzheimer’s disease, Parkinson’s disease (PD) is the second commonest neurodegenerative disorder. In PD, the disease process critically involves nigrostriatal dopaminergic (DA) neurons, resulting in their loss and decreased dopamine levels in the dorsal striatum. For individuals affected by PD, the development of tremor, rigidity and cognitive decline are compounded by an increased risk of early dementia and postural instability. The concept of replacing lost DA neurons with healthy cells of the same lineage underpins one current clinical approach that uses allografts of human fetal ventral mesencephalic (hfVM) tissue. However, many fetuses are required to graft a single individual, owing to the loss of harvested DA neurons denuded of their natural stromal microenvironment.

**Results**

In this study, the authors pioneer the use of nanotechnology to model a surrogate ‘nano-stroma’ engineered to deliver proneural growth factors to targeted cells. They report that LIF-nano-stroma, a prototype biodegradable poly(lactic-co-glycolic acid) (PLGA)-based nanoparticle matrix (PLGA is FDA approved for therapeutic devices) loaded with leukaemia inhibitory factor (LIF; a proneural and reparative factor) provided fourfold increased survival when targeted to DA neurons maintained *ex vivo*. Moreover, human fetal DA neurons pre-coated with LIF-nano-stroma outnumbered cells pre-coated with empty-nano 12 weeks after transplantation into the striatum of nude rats. Notably, DA cells showed the greatest benefit from LIF-nano-stroma pre-coating. The authors also developed a nano-stromal construct using the small molecule XAV939 (XAV) as cargo. XAV is a potent inhibitor of the Wnt–β-catenin signalling pathway, which is involved in neural development. XAV-nano-stroma retained bioactivity and promoted the survival of hfVM *ex vivo*, including that of DA precursors.

**Implications and future directions**

These findings provide the first demonstration that LIF-nano-stroma and XAV-nano-stroma both have pro-survival effects on human DA neurons. Moreover, they show that, to work, the nanoparticles must be attached physically to their target cells and must include a cargo – simple attachment of empty nano-stroma was not beneficial. More generally, these findings have implications for the application of nanotechnology to healthcare. Nano-medicine is a rapidly expanding field, and modelling the outcome of treatment is crucial for its progress. Notably, these findings highlight the potential of using surrogate nano-stromal niches to modulate the fate-determination pathways of precursor cells towards a desired phenotype. This approach might in future be used to support the translation of cell therapies derived from embryonic stem cells or induced pluripotent cells for neurodegenerative disease into clinical practice.

Leukaemia inhibitory factor (LIF) is an IL-6 family member; LIF-specific activity is endowed by the LIF-specific receptor subunit gp190. LIF is attractive as a therapeutic agent in cell therapy for PD, being both proneural and reparative (Laterza et al., 2013; Deverman and Patterson, 2012; Ling et al., 1998) as well as anti-inflammatory and tolerogenic through suppressing IL-6-linked TH17-mediated inflammatory immunity (Gao et al., 2009; Cao et al., 2011). To overcome the problem of the rapid degradation of LIF and its excretion *in vivo*, we previously developed LIF formulated as PLGA-nanoparticles (LIF-nano) and demonstrated that LIF-nano is superior to soluble LIF in being able to provide a stable source of low-dose LIF, mimicking paracrine delivery (Park et al., 2011), with activation of the STAT-3 signalling pathway (Anna Williams and S.M.M., unpublished observations). As such, LIF-nano met our aims to provide stromal support when attached to targeted cells by antibody and has been used successfully in the past for manipulation of T cell lineage differentiation (Gao et al., 2009; Park et al., 2011). Likewise, LIF-nano is known to support mouse embryonic stem cells in the absence of soluble LIF (Corradetti et al., 2012) as well as promote the survival of transplanted pancreatic β-cell islets (Dong et al., 2012). Therefore, we now asked, can a surrogate stroma provided by LIF-nano be targeted to primary neural precursor cells, and, by so doing, increase cell survival? Compared with other cell types, DA cells are highly susceptible to stress but nevertheless are the obvious candidate for neural grafting in PD. Thus, we looked at the response of these cells to LIF-nano and found that it had pro-survival effects on both rodent and human fetal nigral cells, and also *in vivo* for primary hfVM cells transplanted into the striatum of nude rats.

In addition to LIF-nano, we generated and tested a range of other novel nano-therapeutics as stromal candidates, including the small molecule XAV939 (XAV). XAV is a potent inhibitor of tankyrase (Huang et al., 2009), and this inhibition stabilises Axin1 and Axin2. Axin2 is known to bind β-catenin – the mediator of Wnt signalling (Moon et al., 2002; Vacik et al., 2011) – and increased levels of Axin2 are able to retain β-catenin in the cytoplasm, preventing its nuclear translocation where it binds to T cell factors (TCFs) that regulate Wnt-controlled gene expression: these include genes required in neural development (Patapoutian and Reichardt, 2000). We therefore reasoned that the use of XAV-nano might modulate the Wnt–β-catenin signalling pathway and promote plasticity during neural lineage development, with the potential to manipulate lineage differentiation to give more neurons, including, for example, more DA cells, which is especially relevant to future stem-cell-based approaches to treating PD. We now show that XAV-nano retains XAV-mediated bioactivity and is strongly pro-survival when targeted to hfVM-derived cells, including the neural precursors of human DA cells.

## RESULTS

### Neurogenic ‘stromal’ nanoparticles

**Fig. 1.**
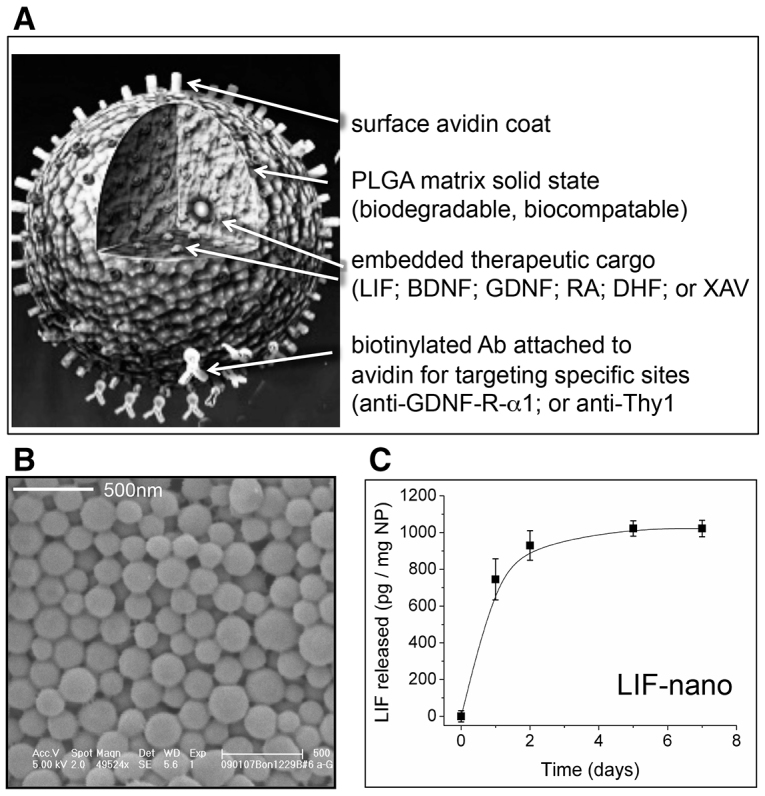
**Design and cargo release rate of nanoparticle constructs.** (A) Cartoon of PLGA nanoparticle functionalised with avidin for binding of biotinylated targeting antibody. Cargo for delivery is embedded within the solid PLGA matrix. Cargo abbreviations of LIF, BDNF, GDNF, DHF and XAV are detailed in the main text; RA, retinoic acid. (B) Scanning electron micrograph of nanoparticles showing tight size distribution: average diameter is 120 nm. (C) Cargo release rate from PLGA nanoparticles (NP) as exemplified by LIF after suspending LIF-nano in aqueous medium and sampling using ELISA measurements.

PLGA formulation of the neurogenic factors was based on the successful LIF-nano construct with proven bioactivity in guiding T lymphocyte lineage differentiation (Fig. 1). In the current study, novel cargo-carrying nanoparticles with the potential to influence neurogenic cell fate were created, including those carrying brain-derived neurotrophic factor (BDNF-nano), glial-derived neurotrophic factor (GDNF-nano), 7,8-dihydroxyflavone (a TrkB agonist; DHF-nano) (Jang et al., 2010) and XAV939 (XAV-nano). Encapsulation of each cargo within the avidin-coated nanoparticles was successful, although GDNF-nano proved to be relatively unstable, requiring several preparations before incorporation with good bioactivity was achieved. Cargo incorporation was around 1/1000 as measured by ELISA so that 1 mg of nanoparticles was estimated to correspond to 1 ng of cargo. In fact, the potency of the nano-formulated growth factor when compared with the soluble growth factor was increased in the order of 100- to 1000-fold as previously shown for LIF-nano (Park et al., 2011) and mathematically modelled by Labowsky and Fahmy (Labowsky and Fahmy, 2012). A fully detailed procedure for creating the PLGA-nano-stromal constructs used in this study is provided in the Materials and Methods section.

### Nano-LIF-stroma is pro-survival for E14 rat DA neurons

**Fig. 2.**
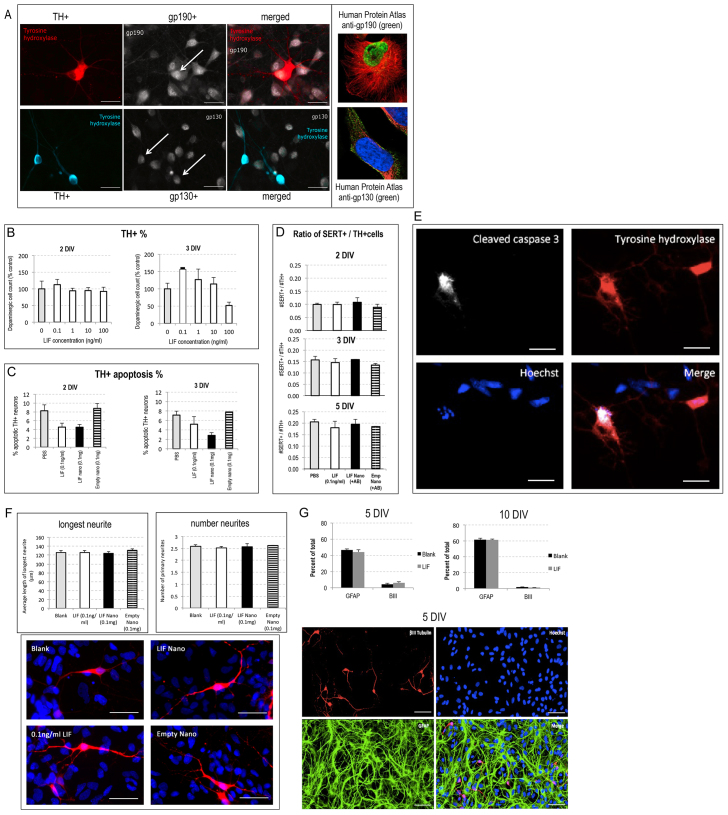
**Rat fetal VM DA cells respond to LIF with increased survival and normal differentiation**. (A) Immunocytochemistry of primary E14 rat VM cells after 3 days in culture in differentiation growth medium: staining for tyrosine hydroxylase (TH) and co-staining for the LIF-R subunits, gp130 or gp190, revealed co-expression in TH^+^ cells. Upper left three panels: TH (red) and gp190 (white) staining showed, unexpectedly, gp190 to be nuclear in the rat VM cells, a location also seen in the human glioblastoma astrocytoma cell line U-251 stained with rabbit anti-gp190 HPA00478 (green, upper right insert). Lower left three panels: TH (blue) and gp130 (white) staining showed gp130 at the plasma membrane; for reference, the human U-2 OS cell line stained with rabbit anti-gp130, CAB025784, is shown (lower right insert). Arrows indicate double-positive DA cells. Scale bars: 25 μm for rat VM cells; 10 μm for human cells. For publically available Human Protein Atlas (HPA) details see http://www.proteinatlas.org/ENSG00000113594/subcellular (gp190) and http://www.proteinatlas.org/ENSG00000134352/subcellular (gp130). HPA stains: green=antibody; red=tubulin; blue=nucleus. (B) Effects of soluble LIF on the percentage of TH^+^ cells in primary E14 rat VM tissue grown as monolayer cultures in differentiation medium supplemented with a range of LIF doses from 0.1 to 100 ng/ml for 2 (left panel) or 3 (right panel) days. At 2 DIV, no significant effect of LIF treatment was seen (one-way ANOVA, F_4,14_=0.31, *P*>0.05); at 3 DIV, 0.1 ng/ml LIF significantly increased the DA cell count as compared with untreated controls (one-way ANOVAs, F_4,14_=4.55, post-hoc Tukey tests, *P*<0.05). (C) Replicate cultures of primary E14 rat VM were treated with soluble LIF, LIF-nano or empty-nano and fixed at 2 DIV (left panel) or 3 DIV (right panel). Apoptotic DA neurons were identified as cells immunopositive for both tyrosine hydroxylase (TH) and cleaved caspase-3. Both soluble and nanoparticle LIF reduced the proportion of apoptotic DA neurons at 2 DIV (one-way ANOVA, F_3,11_=14.13, *P*<0.01, post-hoc Tukey tests, *P*<0.05). No treatments had a significant effect on levels of dopaminergic apoptosis at 3 DIV (one-way ANOVA, F_3,10_=4.33, *P*>0.05). (D) LIF treatment does not alter the serotonergic to DA ratio in E14 rat VM cultures. Monolayer cultures of E14 VM tissue were treated with soluble LIF (0.1 ng/ml), LIF-nano (0.1 mg/million cells) or empty-nano (0.1 mg/million cells). After 2, 3 or 5 DIV, the cultures were fixed and stained for TH and SERT. One-way ANOVA demonstrated that there was no effect of treatment on the serotonergic to DA cell ratio at 2 DIV (F_3,13_=0.45, *P*>0.05), 3 DIV (F_3,10_=0.68, *P*>0.05) or 5 DIV (F_3,12_=0.36, *P*>0.05). Thus, LIF therapy does not alter rat fVM composition. (E) Photomicrographs of a single group of fixed cells co-stained at 2 DIV for cleaved caspase-3, TH and Hoechst to identify apoptosis of TH^+^ cells. (F) LIF treatment has no effect on the morphological development of DA neurons *in vitro*. Monolayer cultures of rat E14 VM tissue in differentiation medium were treated with soluble LIF (0.1 ng/ml), LIF-nano (0.1 mg/million cells) or empty-nano (0.1 mg/million cells). At 5 DIV, cultures were fixed and stained for TH. Representative images of individual TH^+^ DA neurons from each treatment group are shown in red. The development of DA neurons was quantified as the length of the longest neurite and the absolute number of primary neurites per neuron (see histograms). Neither measure was found to be affected by LIF treatment, with the average length of the longest neurite being 125 μm (F_3,12_=0.32, *P*>0.05) and average number of primary neurites per neuron being 2.5 (F_3,12_=0.17, *P*>0.05). Scale bars: 50 μm. (G) Expansion of E14 VM neural progenitor cells in the presence of 0.1 ng/ml LIF did not alter the neuronal:glial ratio. E14 VM precursor cells were expanded as neurospheres for 7 days in proliferation medium containing 10% FCS with, or without, 0.1 ng/ml LIF before plating in differentiating medium for 5 or 10 DIV. GFAP and βIII-tubulin staining respectively identified cells of glial and neuronal lineages: cell counts showed no significant effect (two-way Student’s *t*-tests, *P*>0.05 at either 5 or 10 DIV) as shown in the histograms, and illustrated for 5 DIV staining. Scale bars: 50 μm. Quantification was based on five independent repeats (i.e. five litters of embryos) enumerated as detailed in the Materials and Methods together with the protocol for morphological analyses and statistical analysis.

We first asked, do primary fetal rat E14 ventral mesencephalon (VM) cells, known to include DA precursors, benefit from stromal support provided by LIF-nano? Because LIF signalling requires the heterodimeric receptor consisting of gp190 (LIF-specific subunit) and gp130 (common signalling subunit), we needed to confirm that the tyrosine-hydroxylase-positive (TH^+^) cells co-express gp190 and gp130. Fig. 2A shows adherent cells at 3 days *in vitro* (DIV), with co-staining for TH plus gp190, and TH plus gp130. Unexpectedly, gp190 staining was nuclear. This subcellular stain is seen in the Human Protein Atlas (HPA) database (insert to Fig. 2A). The presence of nuclear receptors could indicate an intracrine signalling capacity for fetal VM cells, as has been shown for arcuate neurons expressing nuclear receptor for the IL-6 family cytokine member ciliary neurotrophic factor (Couvreur et al., 2012).

We next tested responsiveness by adding soluble LIF to primary cultures of differentiating E14 VM cells. After 2 DIV – when the cells had become stably adherent – no effect of LIF was seen but, after 3 DIV, it induced a significant increase in TH^+^ cells (Fig. 2B). Reasoning that increased numbers of TH^+^ cells reflected either increased survival or increased lineage amplification, we looked for early effects on apoptosis by staining for caspase-3 cleavage and here included LIF-nano targeted to GFRα-1, having first confirmed dual staining for GFRα-1 plus ΤΗ. Both soluble LIF and LIF-nano halved the rate of apoptosis seen in the TH^+^ cell population at 2 DIV as revealed by co-staining for cleaved caspase-3 (Fig. 2C,E). Importantly, empty nanoparticles (empty-nano) targeted to GFRα-1 had no effect (Fig. 2C) and extended controls showed that both targeting of the nanoparticles plus delivery of a cargo was required for the nano-stromal effect, as detailed by Dyson et al. (Dyson et al., 2014). Because apoptosis was recorded at 2 DIV, when no difference in TH^+^ cell numbers was apparent, we surmise that some of the TH^+^ cells seen at 2 DIV in untreated control cultures were already in the early stages of apoptosis. The data implies a pro-survival property of LIF for DA precursors, including when delivered as a nano-stroma targeted to GFRα-1.

We also investigated potential effects on neural lineage composition in the context of growth factor delivery during development of precursor cell populations. For example, high serotoninergic to DA cell counts in hfVM grafts in PD patients has been linked to the development of graft-induced dyskinesias (GID). Because serotoninergic (SERT^+^) cells express GFRα-1 (Dyson et al., 2014), we measured the ratio of SERT^+^ to TH^+^ cells in rat fVM cultures. Fig. 2D shows that neither soluble LIF nor LIF-nano altered the serotoninergic to DA cell ratio measured at 2, 3 and 5 DIV. Fig. 2F shows maturing DA cells with equivalent differentiation in the presence of soluble LIF, LIF-nano and empty-nano: this was confirmed by analysing neurite numbers and length.

To address the possibility that LIF might influence glial versus neuronal differentiation, we investigated primary E14 VM tissue expanded in proliferation medium as neurospheres in the presence, or absence, of 0.1 ng/ml soluble LIF. Following a week of expansion, tissue was plated as monolayer cultures in differentiation medium without further LIF treatment and grown for either 5 or 10 days. Quantification of βIII-tubulin-positive neurons and glial fibrillary acidic protein (GFAP)-positive astrocytes (Fig. 2G) demonstrated that LIF exposure during neurosphere expansion had no effect on subsequent neural versus glial differentiation at either of these two time points. The relatively high proportion of glia reflects expansion as neurospheres when precursors decline in the ability to make neurons.

### Nano-stromal-induced activation of Trk-B is pro-survival

**Fig. 3.**
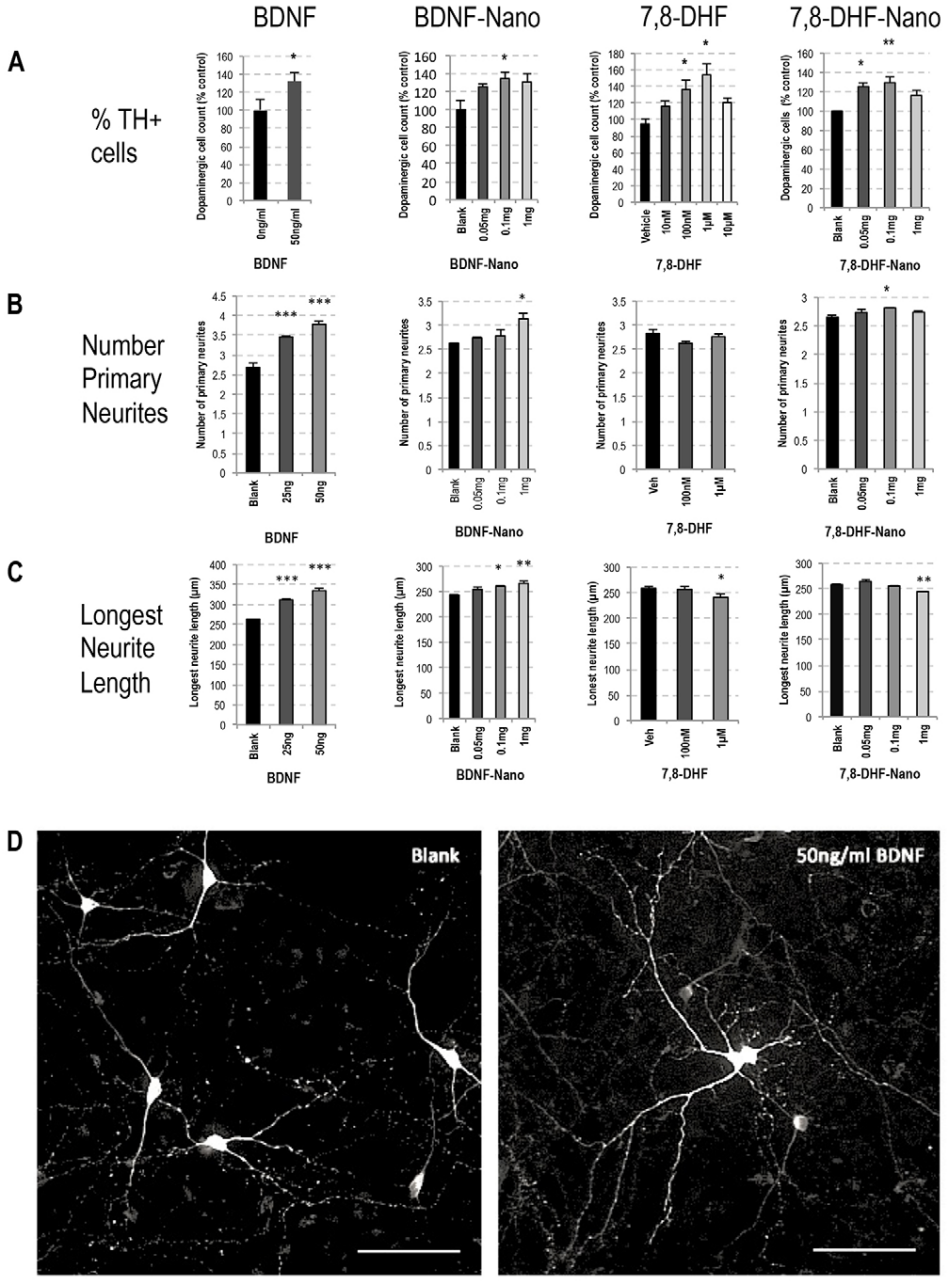
**Comparison of BDNF versus 7,8-DHF, each in soluble and nano formulation, showed effects on rat E14 VM DA neuron survival and maturation.** (A) The sets of histograms show % TH^+^ cells in rat E14 VM cultures at 7 days post-plating in differentiation growth medium. In these same cultures, the histograms in B show the average number of primary neurites per DA TH^+^ cell, and C shows the average length of the longest neurite per TH^+^ cell: results for each set are detailed below. Total cell counts of Hoechst-stained nuclei in each culture were made and the effect of therapy on total cell number is stated in the relevant figure legend text. The four treatment groups were BDNF, BDNF-nano, 7,8-DHF and 7,8-DHF-nano: the overall data are summarised in Table 1. The nanoparticles were targeted to Thy-1. No toxicity was observed in any of the culture conditions. (A) BDNF primary E14 VM cells cultured in 50 ng/ml BDNF showed significantly enhanced survival of the DA neurons (one-tailed Student’s *t*-test, *P<0.05): the total cell count was unchanged (two-tailed Student’s *t*-test, *P*>0.05). BDNF-nano primary E14 VM cells cultured in Thy-1-targeted BDNF nanoparticles at 0.1 mg/million cells showed significantly enhanced DA cell survival (one-way ANOVA, F_3,29_=3.73, *P*<0.05, post-hoc Tukey tests, *P<0.05): the total cell count was unchanged (one-way ANOVA, F_3,18_=0.54, *P*>0.05). 7,8-DHF primary E14 VM cells cultured in 7,8-DHF showed a dose-dependent enhancement of DA cell survival at 100 nM and 1 μM (one-way ANOVA, F_4,27_=3.65, *P*<0.05, post-hoc Tukey tests, *P<0.05): treatment had no effect on total cell count (one-way ANOVA, F_4,17_=0.28, *P*>0.05). 7,8-DHF-nano primary E14 VM cells cultured with Thy-1-targeted 7,8-DHF nanoparticles showed significantly enhanced DA cell survival at doses of both 0.05 and 0.1 mg/million cells (one-way ANOVA, F_3,11_=8.34, *P*<0.01, post-hoc Tukey tests, *P<0.05, **P<0.01): treatment had no effect on total cell count at any of the doses tested (one-way ANOVA, F_3,15_=0.85, *P*>0.05). (B,C) BDNF primary E14 VM cells cultured in BDNF showed a significant increase in the average number of primary neurites per DA neuron (one-way ANOVA, F_2,8_=62.45, *P*<0.001, post-hoc Tukey tests, ***P<0.001) and BDNF also increased the average length of the longest neurite (one-way ANOVA, F_2,8_=51.87, *P*<0.001, post-hoc Tukey tests, ***P<0.001). BDNF-nano at 1 mg of Thy-1-targeted BDNF nanoparticles per million cells significantly enhanced the average number of primary neurites per DA neuron (one-way ANOVA, F_3,11_=6.63, *P*<0.05, post-hoc Tukey tests, *P<0.05) and BDNF-nano at this dose significantly increased the length of the longest neurite in DA neurons (one-way ANOVA, F_3,11_=7.45, *P*<0.05, post-hoc Tukey tests, *P<0.05, **P<0.01). 7,8-DHF shows that, although 7,8-DHF had no significant effect on the average number of primary neurites per DA neuron (one-way ANOVA, F_2,7_=1.74, *P*>0.05), 7,8-DHF at 1 μM significantly reduced the average length of the longest neurite (one-way ANOVA, F_2,7_=8.85, *P*<0.05, post-hoc Tukey tests, *P<0.05). 7,8-DHF-nano shows that Thy-1-targeted 7,8-DHF nanoparticles at 0.1 mg per million cells induced a small but significant increase in the average number of primary neurites per DA neuron (one-way ANOVA, F_3,14_=3.91, *P*<0.05, post-hoc Tukey tests, *P<0.05), whereas 7,8-DHF-nano treatment at 1 mg per million cells caused a significant decrease in the average length of the longest primary neurite (one-way ANOVA, F_3,14_=18.28, *P*<0.001, post-hoc Tukey tests, **P<0.01). Quantification was based on five independent repeats (i.e. five litters of embryos) as detailed in the Materials and Methods. (D) Treatment with 50 ng/ml BDNF enhanced maturation of DA neurons in rat E14 VM cultures. Representative images of control and 50 ng/ml BDNF-treated E14 VM cultures at 7 days post-plating in differentiation growth medium with or without 50 ng/ml soluble BDNF. Tyrosine hydroxylase staining is shown in white. Scale bars: 200 μm.

Because soluble BDNF has been reported to support DA cells (Hyman et al., 1991) and increase the morphological development of DA neurons as assessed by the number and length of their primary neurites (Beck et al., 1993; Kontkanen and Castrén, 1999), we went on to explore the effect of BDNF in nano-formulation on these neuronal features. In contrast to LIF, which signals through the LIF-receptor complex of gp130-gp190, BDNF signals through the neurotrophic tyrosine kinase receptor type 2 (TrkB). It was therefore of interest to include, in parallel to BDNF, a recently published small-molecule agonist of TrkB, namely 7,8-dihydroxyflavone (7,8-DHF) (Jang et al., 2010). We asked: (i) does BDNF-nano retain bioactivity and (ii) is the potency for TrkB signalling comparable between BDNF-nano versus DHF-nano? By determining the percentage of TH^+^ cells as a function of total cell counts, then counting the number of primary neurites per TH^+^ cell and measuring the longest neurite, the effect of signalling through TrkB was recorded as detailed in Fig. 3A–C: this is summarised in Table 1. Fig. 3D illustrates the observed effect of 50 ng/ml soluble BDNF on DA cell maturation. At this dose there was a selective increase in the number of DA cells, to a level that was equivalent to that seen with 7,8-DHF at 100 nM. When in nano-formulation, both BDNF-nano and 7,8-DHF-nano showed maximal equivalent bioactivity at 0.1 mg nanoparticles per million cells. Unexpectedly, 7,8-DHF treatment did not mimic the effect of BDNF in enhancing the morphological development of primary DA neurons (compare the relevant Fig. 3B,C histogram sets). In fact, treatment with 1 μM 7,8-DHF induced a small but significant 7% reduction in the average length of the longest primary neurite from DA neurons. The basis of this effect is unclear.

**Table 1.**
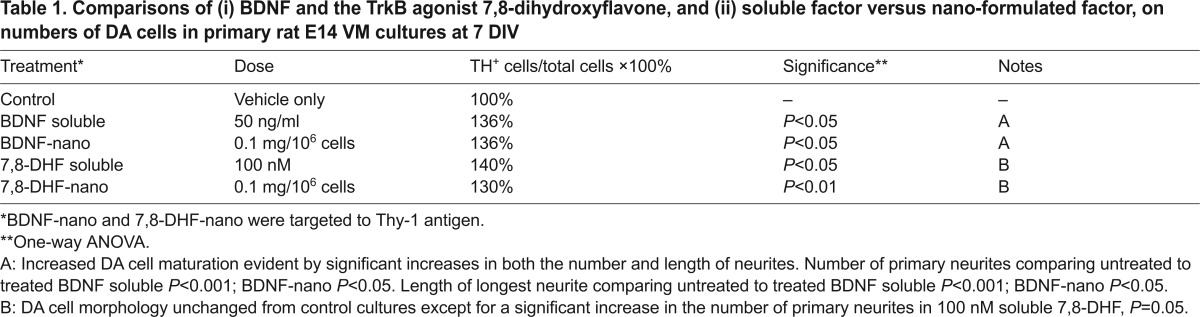
Comparisons of (i) BDNF and the TrkB agonist 7,8-dihydroxyflavone, and (ii) soluble factor versus nano-formulated factor, on numbers of DA cells in primary rat E14 VM cultures at 7 DIV

The BDNF and 7,8-DHF experiments established that different types of cell surface receptor are accessible to nanotherapeutic formulations, and that different types of molecule acting on the same cell surface receptor retain potency in nano-formulation. Of note, the cost of 7,8-DHF is some thousand-fold less than recombinant BDNF – an important consideration in the context of potential therapeutic application.

### hfVM-derived DA cells benefit from nano-stromal support

When preparing donated primary human fetal tissue for cell transplantation into a recipient PD patient, loss of cells – already a scarce resource – is substantial throughout the procedure from harvest to grafting. This means that many fetuses are required to graft a single patient and there is a need to store the fetal tissues at +4°C in hibernation media for several days to allow for the accrual of sufficient fetal material for transplantation. If the yield of DA cells per donated hfVM could be increased, the benefits would be great because fewer donors would be required per patient. Accordingly, we went on to ask, can the nano-stromal approach protect and improve the yield of healthy human DA cells under conditions used for transplantation?

**Fig. 4.**
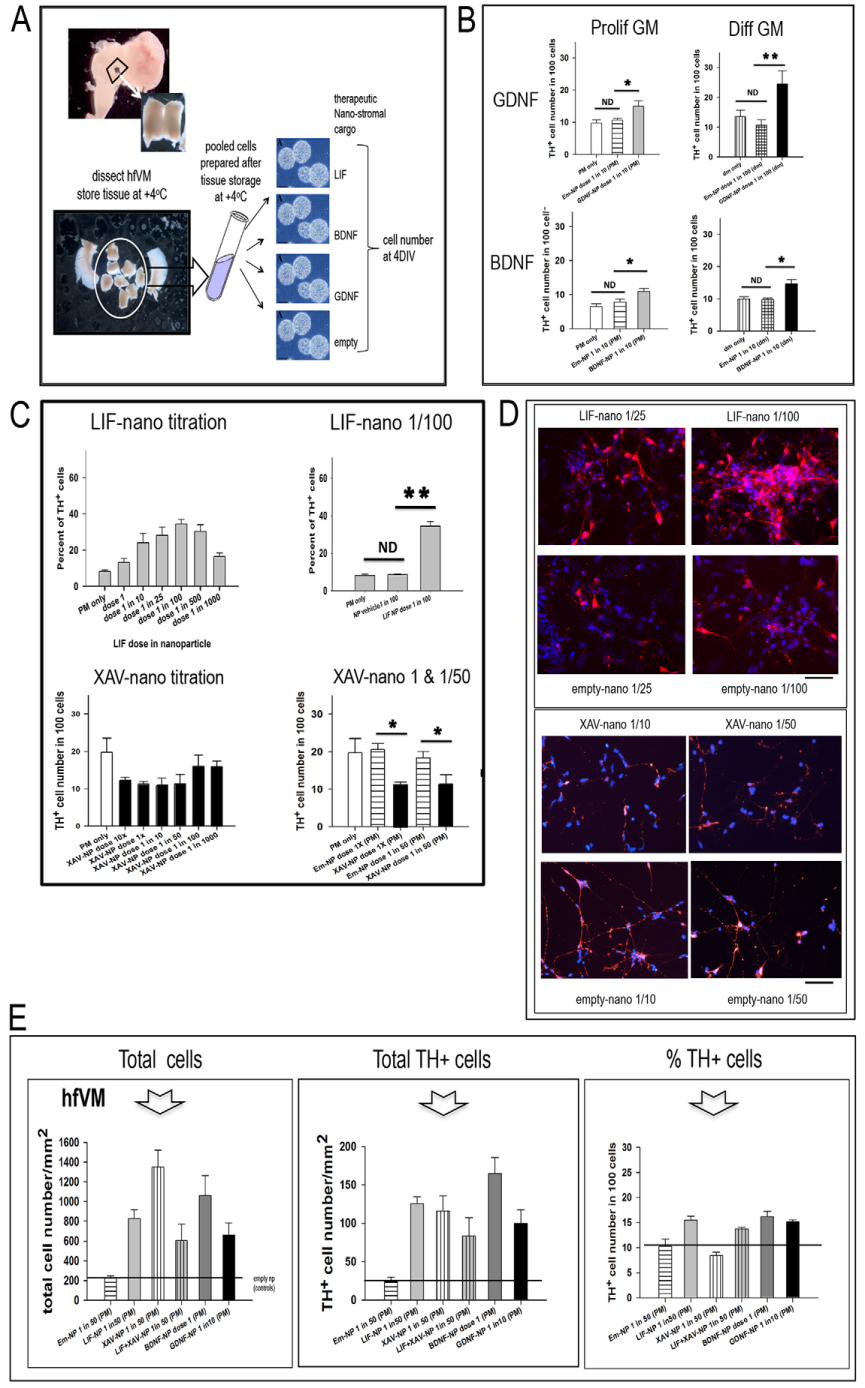
**Nano-stromal-mediated pro-survival effects might be uncoupled from pro-differentiation effects.** (A) Illustrates the procedure for setting up human fetal ventral mesencephalon (hfVM) cultures: the nano-stroma are exemplified for LIF, BDNF and GDNF, with empty-nano as control. hfVM material was obtained at the time of routine termination of pregnancy: fetal age was determined based on crown/rump length. Following dissection, the hfVM was stored at +4°C in HibE for up to 4 days prior to dissociating the cells and coating with nano-stroma. Being of primary human source, no truly identical biological replicates between experiments was possible, but repeat experiments using material from different donors gave similar results for each of the treatments used. Within each experiment there were five replicates per treatment condition. When different treatments were directly compared – as in E – a single batch of donated tissue was used: in some cases cells from multiple donors were pooled prior to aliquoting for treatment to ensure direct comparability. Data are presented as percentage of TH^+^ cells in the total BIII-tubulin^+^ cell population. (B) Histograms showing the percentage of TH^+^ cells in hfVM cultures comparing treatment in either proliferation or differentiation medium (PM or DM, respectively), with or without GDNF-nano (top panels) or BDNF-nano (bottom panels). The experiments shown used either 48-day or 49-day hfVM tissue stored overnight in HibE at +4°C: this was then dissociated into single cells, incubated at 37°C for 16 hours with the various nanoparticle treatments in either PM or DM. The cells were then cultured in DM for 4 days prior to fixing and staining for TH (DA cells) and βIII tubulin (neurons). Both GDNF-nano and BDNF-nano treatment increased the percentage of TH^+^ cells in the culture when compared with empty-nano controls at equivalent dilution, regardless of treatment in either PM or DM. GDNF-nano/PM one-way ANOVA, F_2,11_=5.479, *P*=0.028; GDNF-nano/DM one-way ANOVA, F_2,10_=5.599, *P*=0.030; BDNF-nano/PM GDNF-nano/PM one-way ANOVA, F_2,11_=7.261, *P*=0.013; GDNF-nano/DM one-way ANOVA, F_2,11_=9.862, *P*=0.005. EmNP, empty-nano; ND, no difference; *P<0.05; **P<0.01. (C) Histograms showing the dose response curve based on the percentage of TH^+^ cells in hfVM cell cultures treated with LIF-nano ranging from 1:1 to 1:1000 in PM for 14 hours prior to growth in DM for 4 days (top left). The donor age was 50 days and the VM cells were prepared freshly prior to treatment. Top right compares the LIF-nano dose that had maximum effect (1:100), resulting in a significantly increased percentage of TH^+^ cells compared with untreated controls, or cells treated with empty-nano at equivalent dilution. The lower left histogram shows that treatment with XAV-nano decreased the percentage of TH^+^ cells: in this experiment, the decrease was not significant (*P*=0.060: lower right histogram). The donor age was 42 days and the tissue had been stored overnight in HibE prior to cell preparation and treatment in PM for 18 hours, then culture in DM 4 days. *P<0.05; **P<0.01. (D) Images of the hfVM experiment described in C, comparing the effects of LIF-nano versus empty-nano at equivalent dilution (left panel) and XAV-nano versus empty-nano at equivalent dilution (right panel). Scale bars: 200 μm. (E) Histograms showing total numbers of βIII-tubulin^+^ neurons (left panel), and total TH^+^ cells (centre panel), after the culturing of hfVM cells treated with Thy-1-targeted: LIF-nano; XAV-nano; LIF-nano combined with XAV-nano; BDNF-nano; or -GDNF-nano. The number of TH^+^ cells, as a percentage of the total number of βIII-tubulin^+^ neurons is shown in the right hand panel. *n*=4 in each treatment group where cell counts were taken from five random areas of 1 mm^2^ per coverslip. Error bars show s.e.m.; pairwise multiple comparison procedures post hoc (Holm-Sidak method). Arrow points to the column for XAV-nano-treated cells, where total cell numbers were increased sixfold (left panel), whereas the percentage of TH^+^ cells was decreased (right panel): the overall total number of TH^+^ cells compared with empty-nano controls was fourfold in the presence of XAV-nano. Combined treatment with both LIF-nano and XAV-nano (fourth column) reduced the selective effect of XAV-nano monotherapy on total cell number, and on % TH^+^ cells.

Here, in addition to LIF-nano, we included nano-stromal cargos to target delivery of BDNF, GDNF and XAV, reasoning firstly that BDNF and GDNF are already known to be pro-survival for DA neurons (Hyman et al., 1991; Villadiego et al., 2005), and secondly that XAV might provide in the future a means to modulate neuronal fate. Fig. 4A outlines the process of hfVM harvest prior to cell preparation and nano-stromal treatment. For most of the experiments multiple stored hfVM tissues were dissociated into single-cell suspensions that were pooled prior to aliquoting: each aliquot was coated with nano-stroma targeted to Thy-1 by overnight incubation at 37°C in proliferation medium. Thy-1 was selected as a target antigen because it is expressed on both human fetal DA cells – confirmed in preliminary work here – and on T cells, where LIF-nano is known to be tolerogenic (Gao et al., 2009; Park et al., 2011), a desirable property in the context of cell transplantation. For adherent cultures, the nano-stromal-coated cells were seeded in differentiation medium and cultured for 4 days using five replicate coverslips per treatment group. After staining for TH, βIII tubulin and DAPI, the different cell phenotypes in each culture were counted.

Because proliferation medium (PM) is used to prepare hfVM cells for clinical transplantation, whereas use of differentiation medium (DM) is required for the *in vitro* experiments, we first asked, does the type of growth medium (PM versus DM) influence the response of hfVM cells to nano-stromal delivery of cargo? Responses to the tested nano-stromal types were unaffected by PM versus DM, and Fig. 4B illustrates the results for GDNF-nano and BDNF-nano. Next, the dose of nanoparticle used to provide the nano-stroma was optimised for each cargo type. For LIF-nano, 1/100 dilution had the greatest effect on increasing both the percentage of TH^+^ cells (Fig. 4C, upper panels) and the total cell number. However, unexpectedly, although XAV-nano increased total cell number, the percentage of TH^+^ cells was reduced (Fig. 4C, lower panels). This is illustrated in Fig. 4D. It therefore became of interest to ask, firstly, what was the actual number of TH^+^ cells following XAV-nano and how did these compare to treatment with LIF-nano, BDNF-nano or GDNF-nano? Secondly, is the effect of XAV-nano dominant over LIF-nano, or vice versa? Using a single pool of donor cells, the comparative experiment in Fig. 4E shows total βIII-tubulin-positive (βIII^+^) neuronal cell numbers (left panel), total TH^+^ cell numbers (centre panel) and percent TH^+^ cells (right panel). Only XAV-nano had a selective effect, in that there was a greater increase in total neuronal cell numbers (sixfold) but the percentage TH^+^ cells decreased when compared with empty-nano controls (see arrow in histogram Fig. 4E). In contrast, Fig. 4E shows that LIF-, BDNF- and GDNF-nano each increased both total cell numbers and percent TH^+^ cells. We conclude that XAV-nano treatment might support the increased expansion of neuronal precursors while also offering some support to the TH^+^ cell population. Interestingly, the selective effect of XAV-nano was lost in the presence of LIF-nano (fourth column in histograms, Fig. 4E), where the percent TH^+^ cells was similar to hfVM treated with mono-LIF-, BDNF- or GDNF-nano: this suggests dominance of LIF-induced effects over XAV.

### *Ex vivo* stromal coating of hfVM cells provides long-term benefits *in vivo*

**Fig. 5.**
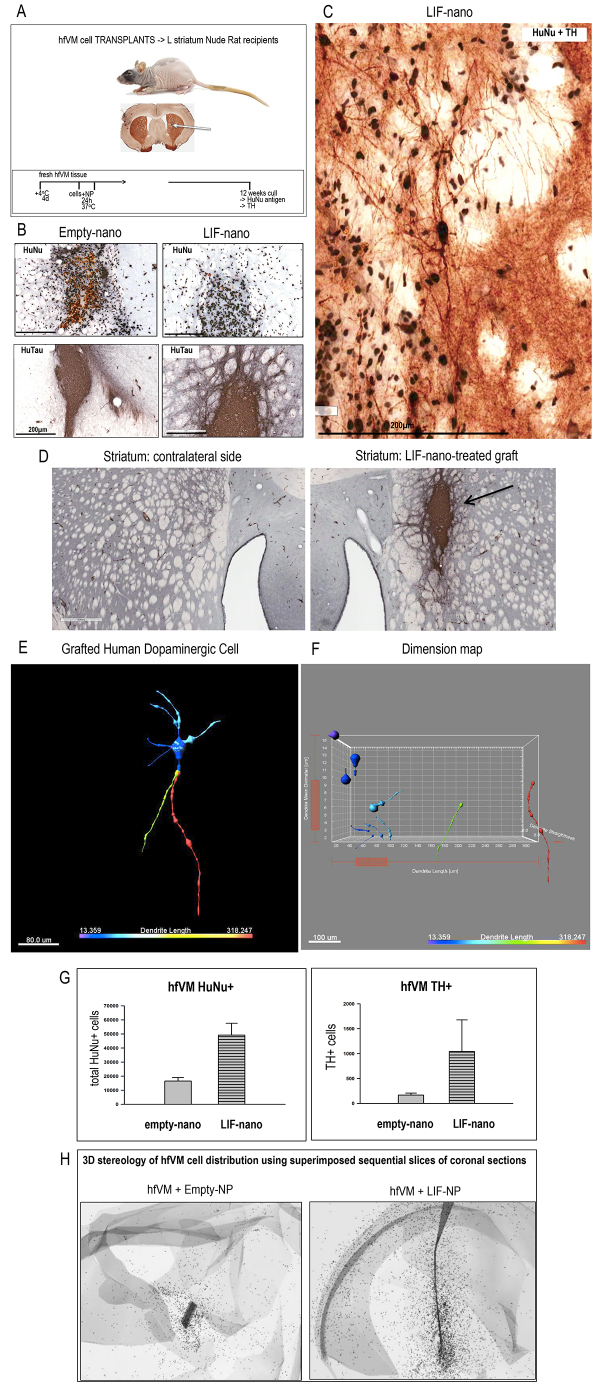
**LIF-nano treatment supports the survival and integration of transplanted hfVM cells into the striatum of the nude rat brain.** (A) Outlines the protocol for transplantation of hfVM cells into the striatum of nude rat recipients following the procedure described previously (Baker et al., 2000). Briefly, hfVM tissue was stored at +4°C in HibE for 4 days prior to cell preparation and treatment with nanoparticles for 24 hours, then grafted into the striatum. At 12 weeks, recipients were culled and coronal brain sections stained. (B) Low-power photomicrographs of recipient striatum 12 weeks post-grafting with hfVM cells treated *ex vivo* with either empty-nano (left panels) or LIF-nano (right panels): sections were stained either for human nuclear antigen (HuNu: black nuclei show living cells, yellow/brown nuclei are dead cells) or for human tau (HuTau, dark grey). Dead cells were more apparent in empty-nano-treated grafts, which also had fewer HuTau^+^ neurites extending within the host tissue compared with the LIF-nano-treated grafts. (C) High-power photomicrograph showing double-positive TH and HuNu human DA cells that have matured in the rat striatum. (D) Low-power photomicrographs comparing graft site with contralateral striatum stained for HuTau. Note HuTau positivity extending beyond the main graft area (arrow). Scale bar: 500 μm. (E) High-power image of a TH^+^HuNu^+^ cell in a graft where the image has been processed using Imaris Filament Tracer software: colours relate to the dimensions of neurites and the cell body. (F) Dimension map of the human DA cell shown in E, using Imaris XT analysis of cell body and neurite diameter, size and shape. (G) Histograms comparing numbers of total HuNu^+^ cells (left panel, *P*=0.058) and double-positive TH^+^HuNu^+^ cells (right panel, *P*=0.362) 13 weeks after transplantation into the striatum of nude rats. Each recipient animal was analysed. Some 2% of the HuNu^+^ cells were DA based on co-staining for TH. (H) Three-dimensional stereology using superimposed sections to show the distribution of HuNu^+^ cells throughout the recipient brain. Cell staining images were taken with a Leica fluorescence microscope with a 20× lens and saved with digital ID. Cells were counted manually using ImageJ software (US National Institutes of Health) with cell counter Plugin. All counting was performed blindly and recorded with ID: total TH^+^HuNu^+^ neurons and total HuNu^+^ neurons were calculated in each recipient animal. For stereology, quantification used Neurolucida 10.

Having established a pro-survival effect for hfVM *in vitro*, we next wanted to know, does coating of hfVM cells with a nano-stroma increase TH cell survival following transplantation into the adult rat brain. Using the xenograft model outlined in Fig. 5A, hfVM cells were prepared as in Fig. 4A, but, instead of being put into culture after coating with the nano-stroma, were implanted into the left striatum of nude rat recipients following the procedures detailed previously (Lopez et al., 2013). Two treatment groups were established from a single stock of hfVM cells, where each recipient received 200,000 cells coated with either empty-nano, or LIF-nano, targeted to Thy-1. Animals were sacrificed at 12–13 weeks, when any increased rate of maturation due to the therapy could realistically first be detected. The brains were removed and sectioned. Fig. 5B shows grafts that had been pretreated with either empty-nano or LIF-nano, stained for human nuclear antigen (HuNu) (upper panels) or human-specific tau (HuTau) (lower panels): LIF-nano treatment seemed to improve the viability and integration of the grafted cells. Fig. 5C shows human TH^+^ cells co-stained for HuNu in a graft pretreated with LIF-nano. Fig. 5D, right panel, shows HuTau expression in a LIF-nano treated graft, where extension of fibres into the host tissue is clear: the left panel shows the contralateral side. Fig. 5E,F show the processed image of a grafted human DA neuron using Imaris Filament Tracer and Imaris XT software to reveal dimensions of the cell body and neurites. The cell counts shown in Fig. 5G revealed a trend for increased numbers of HuNu^+^ cells in the LIF-nano-treated group compared with empty-nano controls, but this failed to reach statistical significance (*P*=0.058; Fig. 5G, left panel). Some 2% of the HuNu^+^ cells were DA based on co-staining for TH, but again the apparent higher numbers in the LIF-nano group failed to reach statistical significance (*P*=0.362). The stereology included recording HuNu^+^ cell distance from the needle tract, where there was evidence for increased migration in the LIF-nano-treated group (Fig. 5H).

Overall, the data suggest that LIF-nano treatment of primary hfVM cells not only supported their survival *in vivo*, but also promoted their integration within the host striatum, with neurites extending well beyond the graft boundary.

## DISCUSSION

Here, we investigated whether the use of a surrogate nano-stroma attached to freshly isolated human, or rat, fetal VM cells promotes their survival by delivery of growth factors or small molecules. Significant benefits were found and two critical determinants identified: (1) the need for a cargo to be delivered: simple nano-stromal cross-linking of cell surface antigen by targeting antibody did not constitute a pro-survival signal in the absence of cargo; and (2) the need to physically attach the stromal nanoparticles to the target cell: unbound cargo-carrying nanoparticles failed to have an effect.

The versatility of using nano-spheres to create a local stroma enables cells to be treated individually, a major advantage for *ex vivo* treatment of therapeutic cells prior to grafting. Notably, we found no adverse effects following treatment of primary embryonic VM cells: indeed the more vulnerable DA population showed preferential survival following nanotherapy targeted to GFRα-1, or Thy-1. The technology platform used to prepare the nano-therapeutic particles is the same as that of BIND Therapeutics (http://www.bindtherapeutics.com), where clinical efficacy and safety are already established: notably, BIND technology is being used in ongoing clinical trials for non-small cell lung cancer, and for prostate cancer.

Our experiments showed that a pro-survival effect can be achieved by nano-stromal delivery of either a biologic or a small molecule, and also when subcellular targets differ. Of the small molecules tested, 7,8-DHF acts at the plasma membrane as an agonist of TrkB; in contrast, XAV acts intracellularly, inhibiting tankyrase enzyme activity. Such versatility of specifically targeted cargos provides potential advantages when considering, for example, a multiplicity of agents to create a favourable niche during transplantation of neural precursor cells. Here, our finding of apparent stromal-derived interactions when XAV-nano is combined with LIF-nano is of interest: firstly, recent work has shown that XAV induces cytoplasmic retention of β-catenin/Axin2 (Kim et al., 2013); and, secondly, evidence suggests that cytoplasmic β-catenin interacts with the LIF-receptor plus E-cadherin to maintain intact cell-cell adhesion and pluripotency (del Valle et al., 2013). Accordingly, we hypothesise that crosstalk between signalling pathways might arise during combined nano-stromal treatment with XAV and LIF. Overall, our data imply that surrogate stromal-induced lineage manipulation might be possible using nano-scale paracrine-type delivery of factors in stromal form to manipulate developmental pathways.

A further point of note was the finding that the pro-survival effect of LIF-nano on TH^+^ cells was detectable in rat E14 VM at 2 DIV, as evidenced by cells expressing cleaved caspase-3, before overt cell death within the TH^+^ population. This suggests that DA cells are especially vulnerable to stress at an early stage of differentiation. *In vivo*, the sustained benefit over 12 weeks gained by a single *ex vivo* treatment of primary hfVM cells demonstrates long-lasting effects, including early integration within the host tissue. These preliminary findings using hfVM support the use of LIF-nano in cell-based therapies, with the added value of tolerogenic properties. Furthermore, conceptually, future developments would include tailored nano-stromal support to provide a surrogate, transient, cell-specific stromal niche: for example, nano-stromal combinations using early delivery of factors linked to embryonic development such as LIF and/or XAV to promote precursor cells transiently, followed by delayed release of BDNF-nano or GDNF-nano, to guide lineage differentiation.

### Summary

Improved survival of DA neurons in cell-based transplantation therapies for PD is needed for this therapy to become a clinical reality. Here, using the highly relevant, stringent model of primary DA cell survival and maturation, we demonstrate that nanoparticles used to create a nano-stroma designed to deliver developmental growth factors, or small molecules, to single cells provides a simple and effective means to support DA cell survival, including primary hfVM cells.

## MATERIALS AND METHODS

### Preparation of PLGA nanoparticles for targeted delivery

Cargos of either recombinant growth factor or small molecule included recombinant murine LIF (Santa Cruz; sc-4378) or human LIF (Santa-Cruz; sc-4988); recombinant human BDNF and human GDNF (R&D Systems); 7,8-DHF (Sigma); and XAV939 (Selleckchem). Cargo was encapsulated in avidin-coated PLGA nanoparticles using a modified version of a previously described water/oil/water double emulsion technique (Steenblock and Fahmy, 2008). Briefly, for example for LIF, 50 μg LIF was dissolved in 200 μl PBS and added dropwise with vortexing to 100 mg PGLA in 2 ml dichloromethane. The resulting emulsion was added to 4 ml of aqueous surfactant solution containing 2.5 mg/ml polyvinyl alcohol and 2.5 mg/ml avidin-palmitate bioconjugate and sonicated to create an emulsion containing nano-sized droplets of polymer/solvent, LIF and surfactant. Solvent was removed by magnetic stirring at room temperature; hardened nanoparticles were then washed three times in deionised water and lyophilized for long-term storage. Nanoparticle size and morphology were analysed via scanning electron microscopy and a Nanosight imaging/sizing system. Release of LIF was measured by incubating particles in phosphate buffered saline (PBS) at 37°C and measuring LIF concentrations in supernatant fractions by enzyme-linked immunosorbent assay (ELISA). Total encapsulation was approximated as the amount of LIF released over a 7-day period and percent encapsulation efficiency calculated as total encapsulation divided by maximum theoretical encapsulation. For cell attachment, biotinylated rabbit anti-GFR-α1 antibody (Acris) or biotinylated anti-Thy-1 was incubated with the avidin-coated nano-particles and free unbound antibody was removed after centrifuging out the nanoparticles, which were then washed once immediately prior to use. Dose was optimised according to dose-response curves (see text): for rat E14 VM this was 0.1 mg/million cells (LIF-, BDNF- and DHF-nano); for hfVM, this was 0.01 mg/million cells (LIF- and XAV-nano) and 0.1 mg/million cells (BDNF- and GDNF-nano).

### Rat ventral mesencephalon cell cultures and staining

Culture medium was either for proliferation (PM), consisting of Neurobasal A (Invitrogen), 2% B-27 supplement, 100 U/ml penicillin, 100 μg/ml streptomycin, 250 ng/ml amphotericin B, 2 mM L-glutamine, 20 ng/ml epidermal growth factor and 20 ng/ml fibroblast growth factor-2, or for differentiation (DM), which was made up of Dulbecco’s Minimal Essential Medium, 10% fetal calf serum, 2% B-27, 100 U/ml penicillin, 100 μg/ml streptomycin and 250 ng/ml amphotericin B.

VM cultures from time-mated Sprague-Dawley rats at E14 followed the protocol described previously (Pruszak et al., 2009). After a single-cell suspension was prepared, the cells were collected as a crude concentrated cell suspension to which the nanoparticles were added and gently mixed. After at least 30 minutes at room temperature, to allow cell attachment of the nanoparticles, the cells were plated on laminin-coated coverslips as 50 μl droplets containing 50,000 cells. After 30 minutes for cell adherence, 500 μl DM was added per well and cultures incubated at 37°C in 5% CO_2_ air. Subsequent medium exchanges involved 50% of the medium being removed and replaced but without nanoparticles. Cultures were fixed for analyses of the total βIII-tubulin^+^ (mouse anti-βIII-tubulin; Sigma-Aldrich; 1/500) and TH^+^ (rat anti-TH; AbCam; 1/300) cell numbers as well as serotoninergic cells (rabbit anti-SERT; AbCam; 1/500) and glia (rabbit anti-GFAP; Dako; 1/500) cells: apoptosis was assessed using caspase-3 release using standard techniques (Cell Signaling Technology). To measure the time course of any therapeutic effects, replicate cultures were analysed after 1, 2, 3, 4 and 5 DIV.

### HfVM cultures and intrastriatal grafting

hfVM was obtained from routine termination of pregnancies at 6–8 weeks of gestational age, subject to full consent being given under full ethical approval. The collected tissues were pooled over a 4-day period, stored at +4°C in Hibernate E (Life Technologies) and processed for culture as outlined in Fig. 3A. Tissue pieces were washed and incubated in HibE/TryplE (Life Technologies) and Pulmozyme (Roche) before trituration, as described previously (Lopez et al., 2013). Specifically, single-cell suspensions were prepared through gentle enzymatic digestion and trituration and were coated with targeted nanoparticles as described for rat VM cells but with treatment in proliferation medium overnight at 37°C. For *in vitro* experiments, nano-stroma-coated hfVM cells were cultured in differentiation medium for 4 days before fixation and staining for HuNu and TH as previously described (Lopez et al., 2013).

For transplantation, unbound nanoparticles were removed by centrifugation prior to transplanting into the left striatum of male Nu-Nu rats aged 12 weeks as described previously (Lopez et al., 2013). Specifically, the cells were collected in PM at a density of 50,000 cells per μl and held at room temperature prior to stereotactic grafting into the striatum, with each graft consisting of 4 μl of the hfVM cell suspension. A common starting pool of cells was used, this being subdivided for nanoparticle treatment, to ensure direct comparison of cargo-related effects. After 12 weeks the rats were culled and the brains removed and processed for immunohistochemistry staining for TH and HuNu. After coronal sectioning, every 6th slice was stained for a given target antigen. Transplant stereology used Neurolucida and cell counting by the Aperio Imagescope according to manufacturer’s instructions to give graft volume, cell counts and the spatial distribution of the grafted cells. Three, and two, recipients received grafts treated with LIF-nano, or empty-nano, respectively: each batch of donor tissue was equally divided between each treatment group to ensure equivalence. Numbers were limited owing to human fetal tissue availability.

Immunocytochemistry of human cells grown on coverslips followed standard procedures. Primary antibodies were diluted in blocking solution and applied overnight at 4°C using rabbit anti-TH (1:1000; Merck Millipore) and mouse anti-βIII tubulin (1:500; Merck Millipore) followed by secondary antibodies for 2 hours at 4°C: antibodies were from Millipore Molecular Probes, including anti-rabbit IgG (A11034, A21206) and anti-mouse IgG (A31570, A21235). Cells were counterstained with DAPI (1 μg/ml). Immunohistochemistry of grafted brains used 40-μm coronal sections collected sequentially into 24-well plates. Sections were taken at regular intervals (for example, 1 in 6) and stained for HuNu (1:1000; Merck Millipore; MAB1281) and TH (Merck Millipore; AB152) using standard protocols. Similarly, a further series of sections were stained for human-specific tau. Each *in vitro* experimental treatment was repeated three times using different donor sources. Within each experiment, five replicate counts/coverslip were made with four coverslips/therapy analysed by ANOVA to give standard error of the mean (s.e.m.).

### Quantification and statistical analysis

Data are presented as mean ± s.e.m. Multiple comparisons in the same data set were analysed by a one-way ANOVA; pairwise multiple comparison procedures were used for post-hoc analysis. Single comparisons to controls were made using a two-tailed Student’s *t*-test. *P<0.05; **P<0.01; ***P<0.001. Further statistical information is provided in the figure legends.
